# Men Tackling Isolating Gender Violence to Fight against Sexual Harassment

**DOI:** 10.3390/ijerph19041924

**Published:** 2022-02-09

**Authors:** Elias Nazareno, Ana Vidu, Guiomar Merodio, Rosa Valls

**Affiliations:** 1 History Department, University Federal of Goiás, Goiânia 74690, Brazil; 2 Sociology Department, University of California, Berkeley, CA 94720, USA; ana.vidu@berkeley.edu; 3 Department Research Methods and Assesment in Education I, UNED, 28040 Madrid, Spain; gmerodio@edu.uned.es; 4 Department of Theory and History of Education, University of Barcelona, 08035 Barcelona, Spain; rosavalls@ub.edu

**Keywords:** sexual harassment, New Alternative Masculinities, bystander intervention, health consequences, isolating gender violence

## Abstract

Scientific literature has shown that both suffering gender-based violence and taking a stand against it could provoke severe retaliation from bystanders, including negative consequences on health. Together with some women, several men—defined as New Alternative Masculinities—have also contributed to fighting against sexual violence in several contexts, also suffering dramatic consequences, known as Isolating Gender Violence (IGV). This article fills the gap on inquiring how men suffering IGV due to intervening in supporting survivors has affected the men’s health. Six in-depth interviews were conducted with men from different contexts and countries and men of different social profiles. The findings reveal how men’s health is better protected when they build networks of support while overcoming the fear of retaliation in achieving to empower direct survivors. In addition, the results recognize men as crucial actors in the struggle against GBV and overcoming IGV, as women potentially do. This may inspire other men to intervene and break the silence regarding GBV in societies and institutions, as it shows that men and women together are needed to fight against GBV.

## 1. Introduction

Isolating Gender Violence (IGV) is defined as the violence, attacks, and retaliation exerted by harassers against people who offer support to gender violence victims (GBV) [1]. Therefore, IGV is not only perpetrated against victims but against both those who stand in solidarity with victims and against direct victims. IGV is perpetuated in order to isolate gender violence victims from their supporters, so they remain isolated and perpetrators may continue acting with impunity [2].

Recent studies have pointed to the fears of reprisals and retaliation as important barriers for bystanders to intervene [3]. What bystanders perceive as possible consequences of their intervention influence their behavior [4,5]. The first study to provide quantitative evidence of the incidence of suffering IGV, as in retaliation and attacks by people who are aware of a situation of GBV, was recently conducted in Spain with 1541 adults. Researchers found that the fear of retaliation was the most frequent reason for not helping victims. 40% of respondents reported not offering help in the case of GBV that they witnessed out of fear of suffering retaliation, consequences, and attacks [6]. Other researches were conducted in this line; the fear of becoming the target of violence and the fear of losing friends were obstacles to intervening among Netherlands teenagers’ cyberbullying witnesses [7]. Likewise, the fear of retaliation, for example, suffering social exclusion, reduces adolescents’ likelihood to intervene in instances of race-based discriminatory behavior [8]. A study of workplace bullying directed at nurses found that fear of possible retaliation if intervening contributes primordially to explain why witnesses hesitate to help victims. This study also found that the absence of fear of retaliation was the main factor that predicted bystanders’ engagement in helping victims [9]. Bystanders that show civil courage are more likely to intervene appropriately despite assuming possible negative consequences for them as a repercussion of their intervention [10]. Another study on neighborhood bystander intervention in intimate partner abuse highlights bystanders’ concern about retaliation from the perpetrator if they intervene. The authors suggest that trust in holding perpetrators accountable is crucial for increasing the likelihood of bystanders intervening [11]. Similarly, to deal with workplace sexual harassment, it has been suggested the importance of protecting workers against retaliation while promoting accountability for perpetrators [12].

Protecting bystanders from retaliation is fundamental for overcoming barriers such as the fear of experiencing IGV and increasing their response. Accordingly, some regional legislation has started to be formulated to tackle retaliations and IGV, aimed at protecting those who protect victims [2]. Nonetheless, to protect bystanders from IGV, it is essential to comprehend the consequences after they intervene, so the impact of these attacks can be identified and measured, contributing to shedding light on a type of violence and its consequences that have often gone unnoticed.

There is limited research on the consequences of bystanders who intervene in situations involving gender violence, sexual violence, or harassment. Research has primarily focused on the health consequences for those who witness violence or bullying. The negative consequences of bullying extend beyond those who are the direct target. Witnessing bullying situations contributes to anxiety and depressive symptoms among middle school students [13]. Despite bystander intervention having been proven a successful strategy for preventing sexual violence, particularly in college communities [14], few studies have examined what happens after bystanders intervene and the possible adverse consequences for them. A study conducted in the USA explored bystanders’ outcomes and the actual consequences of their actions in response to risk for sexual assault. A total of 150 out of 545 surveyed reported having taken bystander action in the past month and qualitatively described their bystander behavior and the responses of those parties involved. The findings show that frequently, victims responded positively to bystanders while perpetrators conveyed negative responses. They frequently responded “upset” or “mad” to bystanders who chose to help victims. Perpetrators also may respond more negatively and with anger if they feel that bystanders blocked their ultimate goal regarding harassing victims [4]. Although this study found that men reported engaging in bystander intervention more than women, the analysis of the consequences of bystander action did not differentiate by gender. Another recent study examined the prevalence and correlates of adverse consequences of bystander intervention among first-year college students in the USA. Approximately one-third of the students surveyed reported having intervened to help someone at risk of sexual assault, relationship abuse, or stalking. A total of 16% to 20% of those who intervened as bystanders indicated experiencing at least one negative consequence, including being verbally threatened (13%), harassed (12%), and even going through severe negative consequences such as being physically hurt (5% to 9%) [15]. The recent studies show that taking a stand against GBV may trigger severe retaliation from bystanders, including negative health consequences.

Thus, as a justification for the study, the research presented in this paper focuses on three very important elements: (1) the IGV happening to men, as usually women are the ones who are the targets in relation to sexual violence and isolating gender violence; (2) the consequences that these attacks and backlashes have on men’s physical and psychological health; (3) how men overcome these negative consequences for supporting survivors, if they do. Drawing on this, the main objectives of this study are the following: to show that men are not only against harassment but are also actively involved in supporting victims; this involvement should not be taken for granted as men experience negative effects on their overall health. In the same way that networks of solidarity are shown to be helpful to support survivors, we were wondering how the men who support victims felt, which features they embraced, and which mechanisms they used to improve their health conditions and feel well, be brave and be in solidarity with most vulnerable people.

## 2. Materials and Methods

### 2.1. Data Collection

This research consisted of a qualitative study through six in-depth interviews with men who had experienced IGV due to supporting victims of sexual harassment or GBV. First, through the six cases analyzed, it was possible to identify the consequences that these men underwent when they intervened in sexual harassment and supported victims and how these consequences affected their psychological, physical health, and well-being in general. Secondly, it has been possible to examine how participants coped with and mitigated the health consequences, analyzing the influence of support networks and the role of New Alternative Masculinities.

The study followed a communicative approach [16] which was selected due to its orientation towards social impact [17], meaning the improvement of people’s lives. The communicative methodology itself has this orientation. Thus, rather that describing a concrete reality, it pretends to provide elements to improve it, so to impact someone’s reality for a better result. This approach promotes an egalitarian dialogue between researchers who bring scientific knowledge and participants who contribute with their daily experiences [16,18]. In addition, the use of qualitative methods has been helpful for investigating bystander-based sexual violence prevention intervention [19]. Moreover, communicative methodologies have been implemented for evaluating violence prevention programs in schools [20] and for the study of New Alternative Masculinities [21].

Interviews lasted between 20 and 50 min. They were conducted through videoconference between November and December 2021 to minimize health risks due to SARS-CoV-2. The interviews were semi-structured. Relying on researchers’ knowledge of different languages, three interviews were conducted in Spanish, two in Portuguese, and one in Catalan. As part of qualitative data collection, participants were asked to explain (a) concrete situations they have intervened; (b) potential situations of harassment suffered because they stood up for sexual harassment victims; (c) how these consequences impacted their health and (d) how they could overcome these effects and move forward. Thus, researchers asked how participants dealt with the negative consequences and what support mechanisms, in terms of network, groups of friends, or other people, they had that strengthened themselves with mutually supportive relationships to endure and overcome IGV. Researchers and participants engaged in an egalitarian dialogue to critically reflect on their past experiences based on the questions generated.

### 2.2. Participants

Six in-depth interviews were conducted with men from Spain and Brazil from different socio-cultural backgrounds, social profiles, and different sexual orientations. The ages of participants ranged from 25 to 69, as shown in Table 1. The four participants from Spain worked at four different universities and were also engaged in social movements. The two participants from Brazil were employed in two different primary and secondary schools.

The finding of suitable participants entailed a methodological challenge due to the issue’s sensitivity. The background of researchers on researching gender-based violence at universities was helpful for finding cases of IGV. Researchers’ expertise on the issue enabled the finding of suitable participants. The testimonies were collected through purposive sampling and contacting networks of alternative masculinities that had activism against gender-based violence. As this is a qualitative study, we were interested in the quality of their arguments, rather than the quantity of them, so we chose people who experienced this reality, in order to identify and present it correctly. The narratives of the people are what gives richness to the study, rather than the number of people participating. Participants were included in the research if they met the criteria of having experienced IGV at some point in their personal and professional careers. The exclusion criteria consisted of not selecting men who had experienced gender violence, but not Isolating Gender Violence. The study specifically looked for those experiencing IGV. The following cases summarize the IGV experiences suffered by participants.

Participant background 1 Joel: He is part of a research group that has supported victims of gender-based violence in universities. The interviewee had also publicly spoken out against harassment at the university, in his department, and at his faculty. The first incident of IGV that he suffered occurred in 2009 when he was finishing his pre-doctoral training period, and his doctoral dissertation was rejected. He was told that it was not an acceptable doctoral thesis to present at the university even though it was a dissertation that met all the criteria for scientific quality. In 2016 there was a second attack when there was an extensive smear campaign in which the victims’ personal lives were attacked for defending survivors of sexual harassment and their supporters. It was a very public attack, in which the defamations and personal attacks were spread even through television, radio, and print media.

Participant background 2 Ricard: Ricard took a clear stand against an adult male aggressor who sexually molested a teenage girl very close to him. When the victim told him what happened, Ricard and another family member decided to believe and support her. However, the abuser reacted, generating a context of harassment, defamations, and hoaxes to discredit the victim’s testimony and those who supported them. As a result, the victim of abuse and the two people who supported her were left alone, and they were socially questioned, not believed due to the lies spread by the abuser. Later on in his career, he was subjected to IGV for belonging to a research group that supported victims of gender-based violence in universities, as in cases 1, 5, and 6. As a result of retaliation and IGV he was not renewed in a university position.

Participant background 3 Tiago: He is a school teacher and a Master’s student researching gender and sexuality. Tiago has intervened to defend people who have suffered sexual harassment, LGTBQ+ discrimination, and racial discrimination while studying at the university.

Participant background 4 Mateus: He is a secondary school teacher. Mateus has defended a female co-worker who was sexually harassed at his school by another male teacher. He reported the incident and offered his support to the victim. In response to his intervention, he experienced IGV. Mateus has also intervened in an episode of homophobic attack against him and another black homosexual friend in the public space.

Participant background 5 Lucas: As in cases 1, 2, and 6, Lucas went through IGV for belonging to a research group that defended victims of gender-based violence at the University. On another occasion, he intervened in a case of sexual harassment against a girl in the metro and was insulted by the aggressor.

Participant background 6 Ivan: He is a university professor who suffered IGV for publicly protecting survivors of sexual harassment. Ivan participated in pioneer research on this issue at his university and also helped the first victim at his school to file a complaint against the professor with most complaints for sexual harassment.

### 2.3. Data Analysis

The six interviews were audio-recorded and transcribed. Following the communicative approach, data was analyzed considering on one hand, those exclusionary elements and barriers, such as the IGV and the adverse health consequences, and on the other hand, the transformative elements that favored the mitigation of the consequences of IGV and the overcoming of it, such as support networks [6].

For the research analysis, we considered the procedure and categories analyzed in the article of Aubert and Flecha [22]. Thus, the discourse analysis method is based on a type of qualitative methodology very sensitive with the environment of the people interviewed. It involves considering not only their narratives but also the context and concrete elements of people’s lives, such as the interactions, experiences, and problems of their surroundings. The triangulation of data has been conducted between researchers, participants, and previous research carried out on the same topic. The relationship established between these three elements is unique. The researcher brings his/her scientific knowledge about the issues and puts it up for discussion with the reality experienced by the interviewee. This dialogue, based on equality and the power of arguments, contributes to the construction of new knowledge, where the voices of the protagonists are included. Table 2 presents a summary of the elements and the categories of the study:

### 2.4. Ethics

The study followed the Ethical Review Procedure established by the European Commission (2013) for EU research, the Data Protection Directive 95/46/EC, and the Charter of Fundamental Rights of the European Union (2000/C 364/01). Informed consent was obtained from the six participants who agreed to participate anonymously, voluntarily, and knowing that their testimonies and data provided would be treated confidentially and only for research purposes. The interviews were coded, and pseudonyms were assigned to participants. Information that could identify the participants and the cases reported were omitted from the verbatim transcription of the quotes. The study was reviewed and approved by the Ethical Committee of the Community of Research on Excellence for All, with the reference number 20211230.

## 3. Results

The results section is divided into three large blocks. The first one refers to the exclusionary elements, which belong to category A of Table 2. Thus, the categories of: suffering retaliation, attacks, IGV; fear of IGV; health and well-being consequences of IGV; psychological health consequences; and physical health consequences, are all included in Section 3.1. of the results as negative consequences of IGV on health and well-being.

The second block, Section 3.2.—building supportive networks to protect male bystander’s health facing retaliation and IGV—involves the first three components of the transformative elements section: receiving support: type, characteristics, and frequency; outcomes of the support received; health improvements of IGV victims. As the New Alternative Masculinities are considered a contribution of this article, the third block—New Alternative Masculinities bystanders overcoming IGV while successfully intervening against sexual harassment—is devoted entirely to this topic.

### 3.1. Negative Consequences of IGV on Health and Well-Being

All participants suffered IGV after intervening in cases of GBV or sexual harassment. The consequences of IGV impacted their health, particularly affected their psychological well-being at the time of the IGV and even in the medium and long term due to the persistence of attacks and retaliation. The most common health and well-being consequences experienced by participants was feeling anxiety, stress, constant nervousness, unrest, concern, insomnia, and lack of control. Frequently, those emotional consequences were combined with other feelings that could be aggravated if those men who acted as bystanders also felt powerless without support from others. For instance, Tiago disclosed that after he took a stand in favor of people who were experiencing harassment and homophobic attacks, he felt fear and anguish partly because of feeling unable to fully defend victims across time due to the difficulties of the case and the lack of support from the institution and other people.

“*I noticed the increase in the level of fear and anguish. But the anguish for not being able, in several moments, to really defend them, because despite the attempt that always existed, sometimes it was not possible to carry out this defense. When I became aware of the situation of these individuals, I really positioned myself in favor of these people… Specifically, I didn’t develop depression, but anguish and fear, yes, in several moments…, anguish more than fear.*”

(Tiago)

Participants were aware that defending victims may mean exposing themselves to possible consequences that might affect their well-being. Even so, these men decided to take a stand in favor of the victims and intervene. Lucas expresses it in the following way explaining first that he intervened in a sexual harassment situation to protect a young woman in the subway; secondly, he narrates the consequences of IGV, such as the daily sadness that he felt as the result of suffering IGV after defending sexual harassment victims at his workplace, the university:

“*The guy in the metro insulted me but I didn’t care. In those situations, you put yourself in a position that affects your well-being at that moment. Then you fear that something is going to happen to you. When it happens to you, you are insulted or attacked; you live with uneasiness, with little insecurity. In a situation I experienced at work, I was pretty much down, from going to work in tears.*”

(Lucas)

#### 3.1.1. Retaliation, Attacks, IGV and Psychological Consequences

Joel explained that after the second IGV attack, he had a solid feeling of helplessness and started to feel anxious. Also, feeling that he was not able to control and manage those negative emotions triggered considerable sadness and an important emotional discomfort. Those concerns, together with lack of support from his family, caused insomnia and tachycardia: “*There was a time when I was having trouble sleeping. I had tachycardia at night, it came together with a delicate family moment and it emerged in this way”.*

The psychological consequences had an impact on the physical health of most participants. As a result of the psychological discomfort and anxiety experienced, participants had physical health problems such as intestinal disorders, headaches, and other psychosomatic manifestations like dermatitis of nervous origin. When bystanders’ physical health is affected, they often do not relate these symptoms to the negative consequences of suffering from IGV. As Joel explains, when he reflected on what was happening to his body, he understood that it was a physical manifestation of the psychological discomfort generated by the IGV. The health affectation, moreover, is aggravated when the attacks and reprisals are prolonged over time:

“*I had a slight anxiety attack and also nervous dermatitis. I associate it because when that happens, at that moment I don’t know how to analyze it, but later on, when I got over it, I think, you realize that you have a whole trajectory of fighting. I guess there is a moment when the body says, “phew, attention!” and I had a small episode of anxiety attacks, dermatitis… and I think they were the explosion of all those moments of tension I lived through.*”

(Joel)

In general, participants had a deterioration in their overall physical health due to the psychological distress experienced due to IGV. It should be noted that the physical health consequences of IGV can be so damaging that they can seriously aggravate other physical conditions or even lead to death. Ivan discloses the case of a close friend who developed cancer metastasis after experiencing ruthless retaliation and attacks due to supporting victims of sexual harassment at his university:

“*These consequences start to be implemented in the body, frequently in the digestive system. The stress, as we already know, damages the brain causing headaches. So, of course, the bad thing about somatization is that when it starts, it is a reflection of what happens in your mind, but then it settles and ends up being, let’s say, diseases of physical type and it can get very serious. It can aggravate cancers as it happened to my friend, it can cause heart attacks, or it can get to extremes and even cause suicide or death.*”

(Ivan)

Ivan described that the first negative consequence of IGV is usually psychological. Receiving attacks, backlashes, or threats directly negatively impacts mental health. Also, the sense of injustice of suffering these threats or attacks for supporting victims affects them psychologically. Additionally, when these attacks extended to other persons close to them and loved ones of the victims of IGV, the psychological impact is intensified because participants realize that the attacks and retaliations are not limited to them, but the IGV is also affecting their loved ones, even affecting children:

“*It affects you because you don’t sleep… because they threaten to kill you at 3 o’clock in the morning… because the feeling of injustice psychologically strains you. But there is a second consequence that affects you much more psychologically: when you see that other people that you love, for example, your family or your kids start to be persecuted for what you have done. This is much harder psychologically than let’s say the first one and creates more problems of conscience. You can be very brave and take the risk and do not care about what happens to you, but, what right do you have to involve minors (on this struggle) such as your sons or daughters? This feeling that it is not only against you but that it affects others whom you love, generates so many psychological problems that lead to somatization.*”

(Ivan)

#### 3.1.2. Fear and Physical Health Consequences

For participants, realizing that the harassment and attacks for supporting GBV victims are not only directed towards them but are also directed against people close to them, such as family members, generates a great concern that has a very high negative impact on their health. Moreover, this psychological distress can be intensified when having been positioned as bystanders is not fully understood by persons close to them, as Ivan illustrates:

“*When someone supports victims as it has been my case in different moments and situations, then it is even less understood, and some people ask you: “why do you get involved in that?”, “do you know for sure that she was not the one who provoked to the harasser”, “but why do you get involved if that not only has consequences for you but also for your family, why do you put them at risk?”… “others don’t do it”… “they will destroy you”… Of course who suffers the most is the victim of direct sexual harassment but another element is added here, that sometimes is not understood by the families or acquaintances: “why are you risking us for something that has not happened to you?”*”

(Ivan)

Undoubtedly, IGV negatively affects those who support GBV victims. With damaging consequences for the psychological and physical health of IGV victims. However, IGV has dramatic consequences for bystanders who offer support to victims, and the attacks can severely extend to bystanders’ loved ones. This also negatively affects bystanders’ health by increasing their suffering and realizing that the retaliation affects their acquaintances.

### 3.2. Building Supportive Networks to Protect Male Bystander’s Health Facing Retaliation and IGV

As evidenced before, IGV negatively affects men’s health. For bystanders who offered support to GBV victims, receiving additional support from others contributed to mitigating and handling the health consequences caused by retaliation and attacks. Support and solidarity are crucial because they help those who experience the consequences of IGV feel better to continue supporting. The type of support is significant. It must involve determined and concrete support, the more and in more diverse settings, the better. Likewise, that victims of IGV receive support is fundamental, so those who support victims of GBV keep supporting them, so they persist despite attacks and reprisals, and as a result, victims of GBV are not isolated and they can become survivors:

“*For victims of isolating gender violence, the only possibility of overcoming the consequences on their physical and psychological health is that they have very strong and courageous support in their different professional and personal environments. No matter how brave and confident a person may be, it is completely impossible that without these networks of real solidarity and support, they will survive the attacks. I am even talking about physical survival. I am talking about death and life. With these networks of genuine solidarity of fraternity, they can be transformed into survivors and persist.*”

(Ivan)

Support to IGV victims can take different forms. Within this support, participants highlight the value of friendship, particularly friendship between men. Lucas explains that real male friends are those who truly support and appreciate you, which gives more strength when dealing with IGV consequences: “*The important thing is to have real male friends. They value you and make you value yourself. That helps. It gives you more strength*” (Lucas). Support and friendship networks help identifying IGV and analyze how perpetrators or harassers act. For example, Ricard took a clear stand against an adult male aggressor who sexually molested a teenage girl very close to him and suffered IGV. For Ricard, receiving support and being able to rely on male friends that helped him to understand that he was experiencing IGV was crucial to face the harassment and support the victim:

“*Luckily, I had male friends who were very clear about what is GBV and abuse. They knew how harassers usually react when victims decide to talk, and that helped a lot to generate a solid position against that situation. Because, of course, you see childhood friends, relatives, who suddenly accepted the version of the harasser… Thanks to these male friends who were clear about what was happening. Being able to talk with male friends about what reactions usually are in these situations, what kind of men use this kind of harassment, grooming, how harassers create a favoring context for them and against the victims so no one believes them… Being able to talk openly about all this helps and strengthens you. That support process makes you value the meaning of friendship and build true friendships with other men.*”

(Ricard)

When the support is organized through formal networks, its capacity for impact and benefit can be multiplied. Participants from Brazil set up a support network for LGBTQIA+ in academia based on solidarity, trust, believing survivors, and building bonds between people who support and help disadvantaged groups and those who suffer injustices, so they do not remain alone. As Tiago explains, this network connects other bystanders and people who need support:

“*I am an academic, so we set up, one year ago, a network of LGBTQIA+ academics, which is the first network in our area of knowledge, the first in the history of the country… Sometimes I am contacted by a person that heard about me. So, sometimes people who are not in the circuit of certain networks appear, but they come up and we end up bringing them into the network, supporting them, and so on.*”

(Tiago)

Regarding the characteristics of these support networks, Mateus points to empathy, its educational elucidative character, its solidarity, and that it is a network of trust that encourages activism:

“*It is a place of open listening, a sensitive listening, a place of non-judgment, and maybe, how can I say that… solidarity is only possible because we recognize ourselves in the process… this solidarity is something that unites us as a group… it’s a place for exchanging and action… it’s not just about listening… it’s also about action, you know?*”

(Mateus)

#### Health Improvements’ of IGV Victims

Men’s supportive networks are crucial to helping them cope and recover from the physical and psychological health consequences of IGV. As Ricard explains, receiving the support from the New Alternative Masculinity network that he belongs to was fundamental for naming and understanding the health consequences of IGV:

“*I am in a network of New Alternative Masculinities. In the group, we talk a lot about health, how important it is to take care of yourself, to be well, even when strong isolating attacks generate a lot of tension, and you don’t know where it comes from. Being able to talk about the consequences helps you understand situations that generate physical or emotional discomfort and that perhaps you are not seeing where they come from. […] You can be suffering a lot in terms of your health. When you see that you are very affected, you are often falling into the consequences of this isolating gender violence. That is why it is so important to talk about it and share it in order to feel better. It is a kind of male solidarity that breaks the consequences of isolating gender violence, isolating yourself from other people, or withdrawing…*”.

(Ricard)

These alternative male solidarity networks help bystanders be better off when they are suffering. They help them compensate for the negative consequences, reduce health effects, and successfully address IGV. For example, Joel explains that after suffering IGV attacks, he quickly received support from a network of NAMs in Valencia. Joel, who throughout his life, at school, and at the university, had experienced homophobic harassment from men of dominant traditional masculinity, seeing that other NAM men gave him support when he underwent IGV was key to improving his well-being:

“*When you realize that there are men who quickly react like these NAM men, I loved it in relation to masculinity, to the visibility of the fact that we can continue to dream about men who are not like those harassers. And it was very useful for me. And this network of friends, of male friends with whom I was able to talk helped me lower the level of tension, relax, and raise my self-esteem. When all this isolating violence arose in 2016, I had to go to Valencia to do a training session with this NAM group and with other people, and I remember they gave such heartfelt applause at the end. It was so deep that it conveyed, “we are here for whatever it takes”. That was a moment that I will always carry in my memory. It shows that friendship, dedication and solidarity can immediately change your emotional well-being. And that day was lovely because I felt that we were not alone. We have a huge network that won’t let us alone.*”

(Joel)

Social support to male bystanders who take a stand with GBV victims shows that people value the fact that other men take a stand against gender-based violence. It is not easy to find people who take a clear stand with victims and act against gender-based violence and sexual harassment. Appreciating and socially valuing that other men of New Alternative Masculinities take a stand and act against GBV contribute to reaffirming bystanders’ commitment. It gives them crucial support in dealing with the health consequences of GBV. Likewise, men who build or participate in supportive networks with other men of New Alternative Masculinities receive greater support that protects men bystanders’ health facing retaliation and IGV.

### 3.3. New Alternative Masculinities Bystanders Overcoming IGV While Successfully Intervening against Sexual Harassment

Some men fight against gender-based violence and sexual harassment. As a result, they suffer from IGV and build or mobilize support networks to address violence and the health consequences of IGV. These men meet the characteristics of the New Alternative Masculinities because they are courageous, stand against gender-based violence, and are against the traditional dominant masculinity that entails aggressive and violent masculinities. These networks of solidarity and support among men of New Alternative Masculinities are characterized by the diversity of the men that it constitutes. They are men of different profiles who help each other and are united in their activism to eradicate gender-based violence. The testimonies explained that some of the participants in these networks are academics, others are working class, and some men work in private companies, including men with different political ideologies, sexual orientation, and gender expression, as well as religious beliefs. This diversity enriches the movement and encourages them to continue the struggle against GBV. As Ricard explains, seeing that the NAM are so diverse helped him to become even more committed to the victims:

“*The worst thing that may happen to a movement that fights against something as widespread as GBV is to be pigeonholed into an ideology, a political party, or a specific progressive profession… There is a huge variety of men in this network, which is very important. In my case, it helped me a lot to see that people with very diverse profiles and positions are very clear about what violence is and what is defamation and what is harassment and what is trying to attack someone…*”

(Ricard)

Men defined as NAMs have a clear vision and an active stance against violence. They hold a proactive attitude and confidence in themselves. They also trust other men defined as New Alternative Masculinities and support each other. Relationships between NAMs who participate in networks such as those described in the previous section create contexts that encourage other men to take a stand in favor of victims and to successfully intervene against sexual harassment as Lucas explains:

“*They encourage you to intervene, not just to be against violence but also to act. In the beginning, it was hard for me. Nevertheless, it was difficult for me because of insecurities and fears, because nobody wants harassers to go against you. What I think makes you take a stand against GBV is to see that there are people who take a stand and the bravery of other people also makes you brave. In my case, it was like that. It’s not that you accept violence, because you never accept it when people attack the weak person, pick on girls… However, taking a stand when those things happen is an important part of seeing that other people also take a stand. If you have referents, role models that help you to position yourself. When you suddenly go on the metro, and you see a guy looking at a girl a lot and harassing her, and you put yourself in the middle, why do you do that? Because you have seen other referents and it is clear to you that this is not right, so you have to look for ways to stop it.*”

(Lucas)

Participants expressed that overcoming the fear of retaliation and achieving to empower direct survivors is important to acting in solidarity and proactively, positioning themselves as effective bystanders despite the potential fear of retaliation and IGV. To do so, they share strategies for bystander intervention and how to protect themselves while intervening and offering support to GBV victims. Participants express that talking and sharing is key for encouraging them to intervene and for addressing IGV as Ricard explains:

“*Yes, we talk about how to intervene as bystanders. The fact of being able to share how to take a stand and protect yourself as much as possible while taking a stand. The key is to be able to talk about it, to be able to talk about it with guys, with male friends, with male colleagues with whom you share a concrete vision of the role of masculinity in the world, like NAM. To talk about what we can do differently from dominant masculinities, the key is active positioning in favor of the victims and talking a lot about what might happen, the retaliation if you take a stand.*”

(Ricard)

Being part of these NAM networks prevents men who intervene as bystanders from being left alone to deal with the consequences of IGV. NAMs value an anti-violence attitude. NAM participants who take part in these groups feel more empowered. Their bravery makes these groups more attractive, inviting other men to want to join in activism:

“*You realize that people want to belong in these groups, and you notice it. When you join a group with people with specific values, you realize that this attracts other guys. In these groups, people are clear about it, transmitting it. They transmit it in the sense that they don’t allow an insult, an offense…, if they don’t allow these behaviors, the rest see that in these groups you are fine, you enjoy being yourself, and they want to be part of it… The more guys want to be part of these networks, the lonelier the violent men will remain.*”

(Lucas)

When faced with sexual harassment and IGV, NAMs react and intervene in solidarity with the direct victims of harassment to overcome situations of violence. Their commitment and intervention break with hoaxes that associate sexual harassment with all men as perpetrators.

“*When Ivan, in a departmental meeting, said that what was happening was harassment, it sets an example to society because society needs men who somehow show that masculinity is not about harassment or keeping quiet. That masculinity is something else, and I think it is key because there are people who say, “harassment has always happened… there have always been harassers or disgusting people”… Well, no! There have also been, and many more, men who do not harass, but we have to make them visible. There have always been aggressors, bullies, but we have to make visible that there have always been men who have taken a stand against violence, and sometimes it remains invisible because the aggressor is seen more, he is more visible.*”

(Joel)

The courageous action of men defined as NAMs who intervene in cases of sexual harassment, despite the consequences of suffering IGV, makes it visible that there are types of masculinities that fight against GBV despite experiencing retaliation and attacks from harassers.

## 4. Discussion

Previous studies have suggested that fear of retaliation hinders bystanders from supporting GBV victims [9]. There is increasing evidence that being aware of the potentially aversive consequences of helping a victim of sexual harassment may discourage bystanders [3,6]. However, despite the importance of this, there are barely any studies investigating the consequences for witnesses who intervene in cases of gender-based violence. The few studies that have analyzed the consequences for bystanders who intervene in situations of sexual harassment or GBV point to adverse effects for them. Perpetrators’ responses can entail threats, anger, harassment, or even physical harm [4,15]. Understanding the adverse consequences for bystanders who intervene and offer support to GBV victims is fundamental for preventing them and effectively addressing sexual harassment while protecting bystanders from IGV. To respond to this literature gap, this study is the first to provide qualitative evidence on the health consequences of isolating gender violence for male bystanders who took a stand and offered their support to sexual harassment victims.

Some studies have examined gender differences in bystander intervention behaviors and also elements such as how the different reactions to GBV cases reveal the different masculinity positions towards gender-based violence. Previous literature suggests that certain features of toxic masculinity and belief in traditional masculinity are associated with less willingness for bystander behavior [23]. On the other hand, evidence highlights that New Alternative Masculinities (NAM) act against sexual harassment, showing solidarity with victims and being active allies in ending GBV [24,25]. Additionally, men who participate in anti-sexual violence activism show proactive bystander intervention behaviors [26,27]. Despite NAM engaging in proactive bystander intervention in cases of sexual harassment also experiencing dramatic consequences, there are no previous studies on how the consequences of suffering IGV affect their health. This study is a pioneer on qualitatively exploring the health consequences IGV has on men who stand in solidarity with GBV victims and fight against sexual harassment. For this paper, six different cases of men who went through IGV after intervening as bystanders in cases of GBV and sexual harassment were analyzed, including their testimonies and experiences. Also, this study focused on the role of networks of support for dealing with IGV among men, overcoming the fear of retaliation, and effectively responding to sexual harassment while empowering survivors. The findings of this study contribute to increasing data on the health consequences for bystanders after intervening in cases of GBV, suffering IGV, particularly in men. It also provides evidence on the crucial engagement of men struggling against GBV and overcoming IGV.

The six participants acknowledge that Isolating Gender Violence had negative consequences for their health and well-being. The psychological and physical health and general well-being of victims of IGV were affected by the direct attacks and retaliation they suffered after they believed and offered support to GBV victims. Most of them experienced psychological consequences, primarily stress, anxiety, sadness, concern, and insomnia. Regarding physical health consequences, at least four of them experimented with a worsening of their physical health due to the psychological distress caused by IGV. Intestinal disorders, headaches, tachycardia, nervous dermatitis, and cancer metastasis have been reported by participants as physical health consequences of IGV. Health consequences for victims of gender-based violence are similar to those of victims of IGV. 

Another study pointed out that the negative consequences of IGV have a direct impact on the health of direct victims of gender-based violence. For these victims, seeing how their supporters suffer attacks and retaliation from their direct perpetrators negatively affects their health and well-being [22]. Similarly, the present study also shows that not only bystanders’ health is negatively affected due to the retaliation and IGV exerted by harassers against them, but bystanders’ health is much more aggravated by seeing the suffering that harassers inflict on their loved ones due to their support to victims. This dimension of additional health negative consequences emerges novelty in this article through the testimonies of participants, not having been previously explored by other research. The attacks and retaliation from harassers against bystanders and their loved ones, including family members and kids, increase the psychological pain and adverse health consequences of IGV. Encouraging bystanders to intervene has been suggested as a strategy to decrease harassment and retaliation [3]. However, if the health consequences for those who intervene in support of victims are acknowledged and bystanders are protected from GBV, it will be possible to increase the response from bystanders against GBV.

Having a strong support network is critical to helping IGV victims cope and mitigate the adverse health consequences. Receiving support encourages male bystanders to continue supporting GBV victims and reaffirms their position and commitment against violence. Either informal, like friends or peers’ support, or formally organized support networks, all contribute to protecting men bystanders’ health. These networks offer strong and courageous support, so bystanders do not remain isolated or suffer the consequences of IGV alone. Likewise, participants acknowledge that support networks helped them to identify IGV and its consequences, take care of their health and discuss strategies to intervene successfully in cases of sexual harassment while preventing and overcoming IGV. The role of toxic masculinity regarding mental health has been previously investigated. Toxic masculinity is associated with reduced mental well-being and less help-seeking behaviors [28], and are harmful to men and those around them [29]. However, research has shown that men’s health improves when they participate in egalitarian groups that foster alternative masculinities and friendship relationships [24]. This study also shows that male NAM support networks improve the health of victims of IGV, because they care about the health and talk about taking care of one’s health and well-being. As participants show, this attitude and dialogue around health between NAM improve male bystanders’ emotional well-being and physical health.

Previous studies have suggested that adherence to traditional masculinity reduces bystander intervention behavior [23]. On the contrary, research shows that New Alternative Masculinities are active allies against GBV [24,25]. Also, men involved in anti-sexual violence activism show proactive bystander intervention behaviors [26]. The cases of the six participants analyzed for this paper contribute further evidence and make it apparent that there are men of New Alternative Masculinities who act as bystanders overcoming IGV while successfully intervening against sexual harassment. Their testimonies prove that despite the existence of a social discourse that focuses the attention on violent men, other men with NAM characteristics take a stand against IGV and GBV, build and join in support networks. Some characteristics of the NAM highlighted by participants are courage, trust, confidence, solidarity, belief in victims, active listening without judgment, bravery, and taking a stand against violent and dominant masculinities. In addition, they are men who stand in solidarity with women and victims, creating new spaces for dialogue and coordination that enable joint overcoming of unequal relations of discrimination and oppression experienced by women.

There are a few strengths and limitations to note in this study, together with suggestions for future research. The first limitation is the potential bias of self-reported data from participants and how they remember and select the negative experiences and health consequences of IGV in the interviews. Secondly, although the sample was diverse regarding nationality, age, sexual orientation, and gender expression, the study relies on the testimonies from six participants. Previous studies have suggested the importance of including underrepresented groups when analyzing the consequences of bystander action as social status may influence these consequences [5]. This research increases diversity in the research on bystander behavior, including three testimonies from underrepresented groups in universities and schools. However, future research should look for larger samples of IGV victims with other social or cultural characteristics. Also, the findings from the present study can contribute to the design of quantitative research indicators that gather more information on the impact and health consequences of IGV. Thirdly, the difficulties for finding participants willing to share their IGV victimization experiences openly and how their health was affected should be acknowledged. The sensitive topic of research and the possible fear that participants might feel for suffering further IGV consequences if they share their testimonies highlight the relevance of this study.

The findings from this research have important practical implications for bystander intervention. Results might inform future bystander training programs, which should consider the protective health factors that mitigate the consequences of IGV on bystander men’s health. Also, bystander intervention awareness campaigns should acknowledge IGV prevention and foster policies and regulations that effectively protect those who intervene, offering their support to GBV and sexual harassment victims.

## 5. Conclusions

Despite the fear of retaliation and adverse consequences having been suggested as significant barriers for bystanders’ intervention and the relevance of addressing this issue for increasing social support toward victims, few incipient studies have started to analyze the consequences experienced by bystanders after the intervention. Moreover, no studies have specifically looked at the health consequences for men, a gap that hinders its identification and diagnosis. This study shows that suffering IGV retaliation has direct consequences with a negative impact on the psychological and physical health of those who support victims of sexual harassment and GBV. The negative health consequences for male bystanders who experience IGV are exacerbated when their loved ones undergo attacks and retaliation.

Taking a stand and intervening against gender-based violence often involves experiencing IGV. All study participants suffered negative consequences on their health and well-being at different levels. Coping with and overcoming the health consequences of IGV is possible thanks to networks of support and solidarity for those who take a stand with the victims. Particularly important are the support networks and friendships between men of New Alternative Masculinities. The attitude and characteristics of NAMs prevent the health consequences of IGV and isolation while serving as social referents for other men to intervene in cases of sexual harassment, which might contribute to the eradication of GBV.

## 6. Further Research

Further lines of research should compare the health consequences of men and women on supporting survivors, as these may have specificities among genders. Once it has been found that solidarity networks and men’s groups help to improve health outcomes, one could inquire about how these networks are created, what characteristics they have, and what elements make them last over time, if that is the case. Further research could also consider how different racial and cultural backgrounds impact IGV.

## Figures and Tables

**Table 1:** Participants.

Number	Country	Age	Profession	Pseudonym
1	Spain	35–40	University professor	Joel
2	Spain	40–45	University professor	Ricard
3	Brazil	25–30	Schoolteacher	Tiago
4	Brazil	30–35	Schoolteacher	Mateus
5	Spain	40–45	University professor	Lucas
6	Spain	65–70	University professor	Ivan

**Table 2:** Summary of the elements and the categories of the study.

Elements	Categories
A. Exclusionary elements	A.1 Suffering retaliation, attacks, IGV A.2 Fear of IGV A.3 Health and well-being consequences of IGV A.4 Psychological health consequences A.5 Physical health consequences
B. Transformative elements	B.1 Receiving support: type, characteristics, and frequency B.2 Outcomes of the support received B.3 Health improvements of IGV victims B.4 New Alternative Masculinities intervening as bystanders

## Data Availability

The interviews were recorded and transcribed. They are anonymized and safety stored. Only the authors who conducted the interviews have access to this material in order to keep confidentiality and privacy of the participants.
